# Supplementary material for: The Methylation Patterns and Transcriptional Responses to Chilling Stress at the Seedling Stage in Rice
Source: Int J Mol Sci. 2019 Oct 14;20(20):5089. doi: 10.3390/ijms20205089 (PMC6829347; doi:10.3390/ijms20205089)
Supplement: Supplementary file 1 [file ijms-20-05089-s001.pdf]

Table S1. Comparison of Survival Rate of Three Rice Varieties(P427、Nip and 9311) after Low Temperature Treatment in Germination Period.

Variety	Total	After CT (3d/2-3 °C)		After GR (14d/25-28 °C)	
		Survival	Survival rate(%)	Survival	Survival rate(%)
**P427**	**50**	**50**	**100**	**44**	**88**
**Nip**	**50**	**50**	**100**	**32**	**64**
**9311**	**50**	**50**	**100**	**14**	**28**

Note. The values are the means of 3 replicates.

Table S2. Statistics of the reads and mC percent from MedIP-seq of Nip、9311 and P427.

		Raw reads	Uniquely mapped	Mapping ratio(%)	Gene numbers involved	mC (%)	mCpG (%)	mCHG (%)	mCHH (%)
P427	ck	52333888	35513630	67.86	54327	25.69	40.54	26.32	33.14
	3°C	68180724	49906359	73.20	54716	21.83	41.87	27.08	31.05
Nip	ck	65502595	49855090	76.11	56118	23.22	40.88	26.49	32.63
	3°C	64061754	49789115	77.72	55973	24.08	41.28	27.38	31.34
9311	ck	64055270	32746542	61.12	49032	25.33	40.97	26.88	32.15
	3°C	68602860	35550484	61.82	49281	21.19	41.93	27.19	30.88

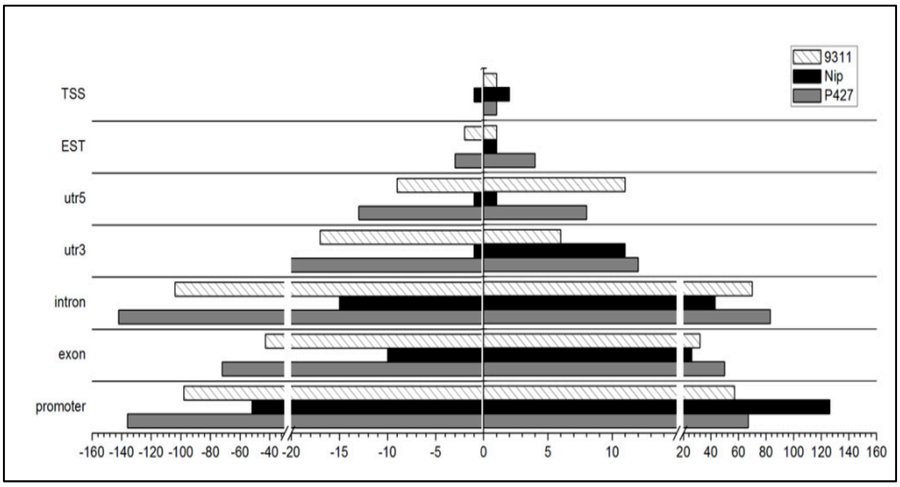

Figure S1. Methylation distribution of cold-sensitive genes in different gene regions.

Table S3. Methylation type(CG，CHG，CHH) distribution of 292 cold sensitive genes in the promoter region.

	CG(%)	CHG(%)	CHH(%)	Total
P427	64(31.68%)	85(42.08%)	53(26.24%)	202
Nip	40(22.35%)	92(51.40%)	47(26.25%)	179
9311	51(32.91%)	49(31.61%)	55(35.48%)	155

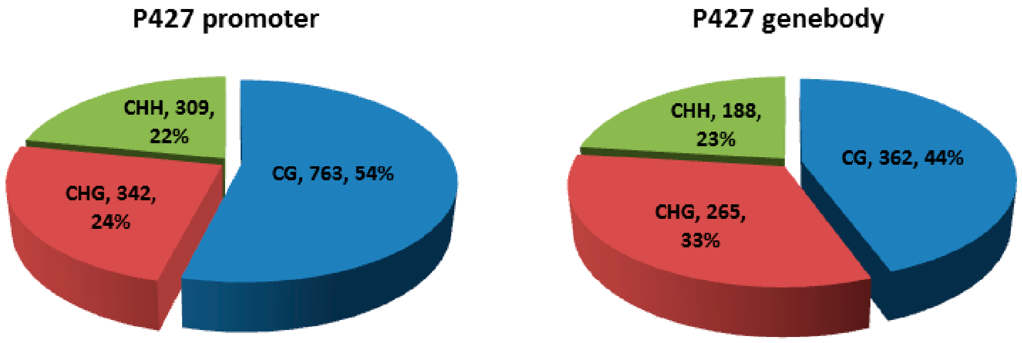

Figure S2. Promoter region and gene body region methylation type distribution in P427.

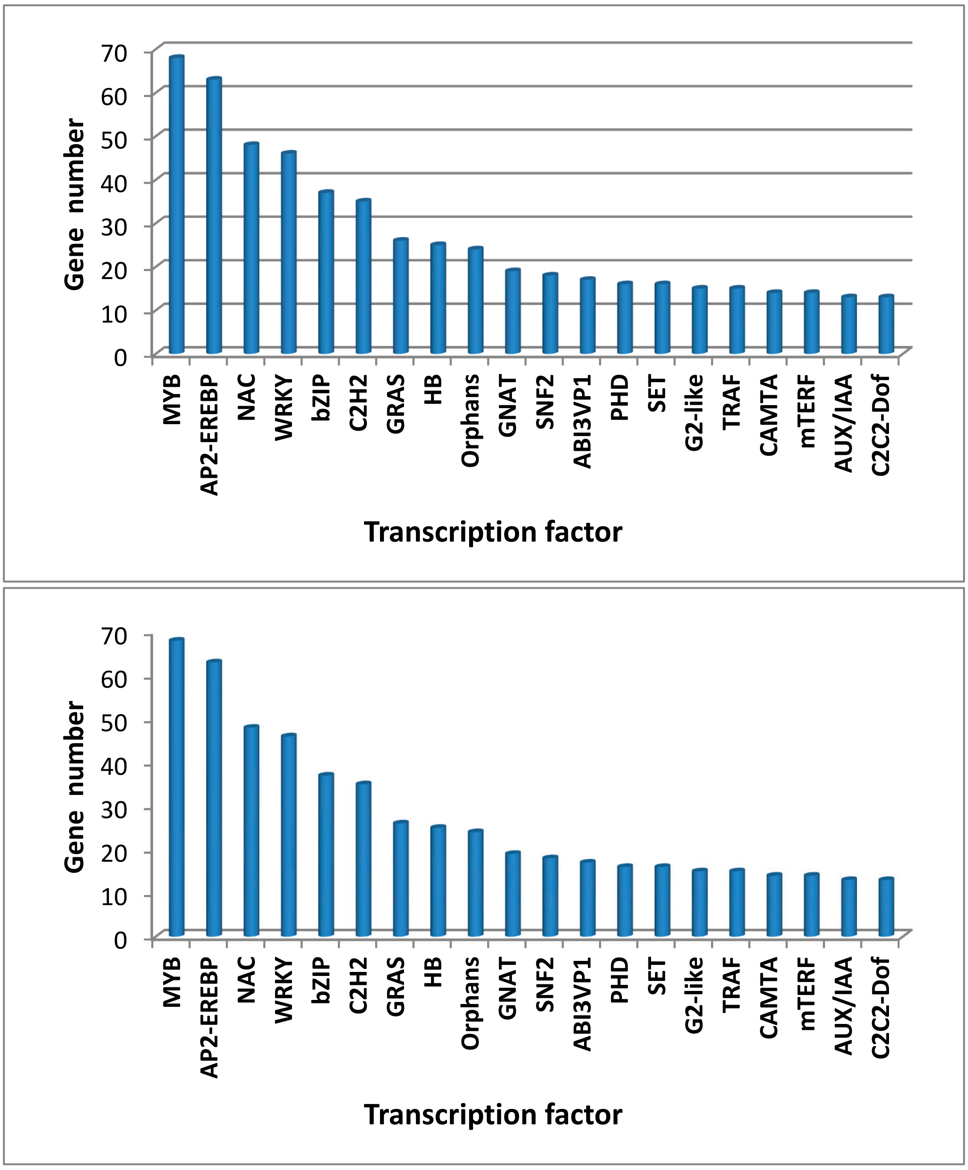

Figure S3. Transcription factor family classification of P427 specific differentially expressed genes.

Table S4. The relationship between methylation and gene expression in 51 genes.

	DNAmeth up	DNAmeth down
DGE up	18	3
DGE down	26	4

Table S5. Primer sequences of PCR

Gene	Forward Primer	Reverse Primer
MPK3 (Os03g0285800)	*GCTCCAACCAAGAACTGTC*	*AGTCGCAGATCTTGAGG*
CRPK1 (Os04g0619400)	*AAGAAGAAGAACAATTTGGCCG*	*TCCTTCTCCTCTTTCCTCCTAA*
OST1 (Os03g0610900)	*GTGAATTCTAGCAAGCAGACAG*	*ACATGTATCCACTTCCCAGTTT*
BTF3s (Os10g0467600)	*TGGAAGAGAAGAAAGAGTCGTC*	*CGTAAAACTCAATTCCACCGTT*
CBF3 (Os08g0545500)	*GATGACGACGTATCGTTATGGA*	*TACACTCGTTTCTCAGTTTTACAAAC*
WRKY7 (Os05g0537100)	*CATCTCCGAGTTCTTCTTCGAC*	*CGTCAAGAATCTCGATCTCTGA*
WRKY70 (Os05g0474800)	*ACATGTGTATGTGGAGTGAGTT*	*TTTTCTCGTTCTCCCTATACGC*
Os06g0638900	*ACCCTACTTTTGACGAGAAGTT*	*AATCACCAAATGGGCGTAATTG*
Os04g0497000	*GACATCTACTTCGAAAACGTGG*	*AGGTTGTACTGCGAGATCATC*
Os10g0463200	*GTGTTAACCGTGTTATGGCAAG*	*TTAATTTCCTCGTGCGAGAGTA*

Table S6. 51 Genes information table

NO.	Gene_id	log2FoldChange	Up/Down	Diff.Methy	Up/Down	C_context	Region
1	Os08g0359100	1.037	up	0.7377	up	CG	promoter
2	Os05g0433900	-3.666	down	0.7040	up	CG	promoter
3	Os09g0468000	-5.01	down	0.6392	up/up	CG/CG	intron
4	Os12g0615500	-1.549	down	0.6280/0.3890	up/up	CG/CHG	promoter/promoter
5	Os01g0962200	-1.169	down	0.5237	up	CG	intron
6	Os09g0237600	-1.352	down	0.5167	up	CG	intron
7	Os06g0126500	-2.376	down	0.4693	up	CG	promoter
8	Os09g0347900	-1.263	down	0.4607	up	CG	promoter
9	Os06g0720000	-1.278	down	0.4548	up/up/up	CHG/CHG/CHG	exon/utr3/TES
10	Os03g0262000	1.919	up	0.4428	up	CG	promoter
11	Os08g0566700	-1.657	down	0.4359	up	CG	promoter
12	Os03g0715600	4.748	up	0.4217	up	CG	promoter
13	Os03g0124100	5.835	up	0.4117	up	CG	promoter
14	Os09g0519100	-2.468	down	0.4078	up	CG	promoter
15	Os06g0701400	-1.137	down	0.4075	up	CHG	promoter
16	Os07g0251200	1.186	up	0.398	up	CG	promoter
17	Os11g0691800	4.425	up	0.3954	up	CHG	promoter
18	Os10g0507600	-1.205	down	0.3836	up	CG	promoter
19	Os08g0539700	-1.966	down	0.3808	up	CHG	intron
20	Os04g0378200	1.558	up	0.366	up	CHG	promoter

21	Os01g0735500	1.573	up	0.3368	up	CG	promoter
22	Os05g0529600	2.843	up	0.3293	up	CG	promoter
23	Os04g0283001	-1.527	down	0.3283	up	CHG	promoter
24	Os04g0497700	-1.447	down	0.3051	up	CG	promoter
25	Os01g0689900	1.269	up	0.3005	up	CG	intron
26	Os08g0411900	1.775	up	0.2941	up	CHG	promoter
27	Os03g0824000	-1.256	down	0.2805	up	CG	promoter
28	Os10g0457400	2.382	up	0.2484	up	CHG	promoter
29	Os04g0585100	1.498	up	0.2397	up	CG	intron
30	Os01g0663800	1.255	up	0.6067/0.2386	up/up	CG/CG	promoter/intron
31	Os03g0675000	-2.411	down	0.2179	up	CG	exon
32	Os04g0637300	5.438	up	0.2156	up	CG	promoter
33	Os03g0232500	-1.303	down	0.2128	up	CHG	intron
34	Os08g0398700	-1.235	down	0.1721	up	CHG	intron
35	Os05g0214100	-1.09	down	0.1658	up/ up	CG/CHG	promoter/promoter
36	Os01g0369200	-2.717	down	0.1549	up	CHH	intron
37	Os02g0278400	1.757	up	0.1252/0.1186	5up	5CHH	2exon/2utr3/TES
38	Os10g0512700	-1.06	down	0.1105	up	CHH	promoter
39	Os02g0331200	-1.977	down	0.0905	up	CHH	promoter
40	Os09g0129800	-2.465	down	0.0836	up	CHH	promoter
41	Os07g0200900	-1.087	down	0.0787	up	CHH	promoter
42	Os07g0258501	-6.552	down	0.0615	up	CHH	promoter
43	Os07g0535700	1.390	up	0.0516	up	CG	promoter

44	Os03g0610900	2.1472	up	-0.6326	down	CG	promoter
45	Os02g0513700	4.822	up	-0.0775	down	CHH/CHH	promoter/promoter
46	Os09g0459800	-1.145	down	-0.0843	down	CHH	intron
47	Os10g0101000	2.789	up	0.6187	up/up	CG/CHG	promoter/promoter
48	Os02g0752300	2.972	up	-0.1131	down	CHH	promoter
49	Os04g0290000	-1.068	down	-0.1535	down	CHH	promoter
50	Os03g0699400	-1.249	down	-0.1840	down	CG	promoter
51	Os02g0652550	-3.685	down	-0.1965	down	CHH	intron

Note. 51 common genes of P427-specific differentially expressed and hypermethylation differentially expressed in the promoter region.
